# Outcomes of professional development activities for selected Texas school personnel helping students cope with behavioral and mental health issues

**DOI:** 10.1038/s41598-023-37298-4

**Published:** 2023-06-26

**Authors:** Gary Wingenbach, Taniya J. Koswatta, Josephine Engels, Jamie Freeny, Sana Haddad

**Affiliations:** 1 Texas A&M University, 2116 TAMU, College Station, TX 77843-2116 USA; 2 Center for School, Behavioral Health at Mental Health America of Greater Houston, Houston, USA

**Keywords:** Health services, Public health

## Abstract

School personnel help youth cope with life and school stressors. However, help may depend on each person’s confidence or knowledge of such issues. In 2019 and 2020, more than 13,800 Texas educators participated in Emotional Backpack Project (EBP) training to support youth coping with behavioral and mental health issues. Post-intervention results revealed significant gains in self-perceived understanding of students’ behavioral and mental health issues, improved confidence in approaching students, parents, or other school staff to discuss students’ harmful behaviors, understanding of mindfulness activities, and increased knowledge of trauma informed schools and trauma informed educators. Teachers and other school personnel were less confident in approaching parents or guardians to discuss youth mental health issues than in approaching students, counselors, and other staff. School personnel’s knowledge, perceptions, and confidence to help students cope with behavioral and mental health issues was significantly better after EBP interventions. EBP training should be adopted widely and occur more than once annually.

## Introduction

Among youth (10–14 years old), suicide is the second leading cause of death and for those 15–24 years old, it was the third most common form of death in 2020^1^. About 93% of Americans believe suicide is preventable^2^. One study^3^ found minimal evidence that suicide can be predicted or preventable at clinical settings. Paris noted that population-based suicide prevention strategies such as restricting access to fatal means, depression screening, and educating gatekeepers provide strong evidence for suicide prevention^3^. Schwartz-Lifshitz et al. concluded that public education, gatekeeper training, and primary care physician education were effective prevention strategies for suicide prevention^4^. Wyman et al. concluded that gatekeeper training for secondary school staff increased staffs’ knowledge of risk factors for suicide and suicide identification behaviors^5^.

### Preventing youth suicide and gatekeeper training

Psychiatric factors commonly influence suicidal behavior^6^, while positive mental health reduces suicidal ideation and suicide attempts^7^. However, most who die by suicide did not seek mental health support before attempting suicide^3^. Thus, gatekeepers (family members, friends, educators, and social workers) could be positive forces countering suicidal ideation for those at risk. For youth, early identification is crucial in preventing suicide; parents and teachers are frontline gatekeepers who can identify and intervene in such circumstances^8^. Kern et al.^9^ noted “evidence suggests that school-based mental health services (SBMHS) have the highest likelihood of reaching youth in need” (p. 205). Are educators prepared adequately to help students with behavioral and mental health needs?

There is evidence^10–12^ that school personnel gain knowledge about students’ behavioral and mental health issues from interventions and training. Jorm et al.^13^ found South Australian teachers increased their knowledge and changed their beliefs about treatment of students’ mental health issues after training. Similar knowledge gains occurred among U.S. mental health professionals after training in suicide prevention^14^. However, Anderson et al.^15^ found limited evidence that mental health training programs improved teachers’ helping behavior or students’ mental health. Burnette et al.^16^ found limited evidence on the impacts of suicide prevention training that led to changes in gatekeepers’ beliefs or attitudes about suicide prevention. Torok et al. concluded there was no relationship between gatekeeper training and recognizing and/or responding to youth at risk of suicide; however, such training increased gatekeepers’ awareness and knowledge of youth at risk of suicide^8^.

Both Torok et al.^8^ and Anderson et al.^15^ found training activities on mental health and suicide prevention are tailored toward teachers more than for other school personnel (e.g., principals, counselors). We identified school-based mental health professionals and other personnel (e.g., coaches, librarians, nurses) are gatekeepers for youth with behavioral and mental health needs. For example, school-based mental health professionals and school administrators were significantly more concerned about students’ mental health than were teachers^17–19^ supporting the notion that school personnel are under-utilized in promoting positive behavioral and mental health in schools. A need exists to train school personnel to support students’ behavioral and mental health development and to determine if such training is effective.

### Emotional backpack project (EBP)

Emotional Backpack Project was developed by the Center for School Behavioral Health (CSBH) at Mental Health America of Greater Houston (MHAGH) in response to Texas legislation^20^ requiring school districts to provide school personnel with training in mental health intervention and suicide prevention. EBP increases educators’ awareness of youth mental health issues and available resources for effective response to youth behavioral matters. The CSBH initiated the EBP in selected Texas school districts to help educators address youth trauma from events such as Hurricane Harvey. EBP objectives are to increase in awareness of suicide warning signs and increase in awareness of resources for help related to suicide risk.

Burnette et al.^16^ identified four factors that influence gatekeepers’ decisions to intervene when people are at risk of suicide: knowledge about suicide, beliefs and attitudes about suicide prevention, reluctance to intervene (especially stigma of mental illness), and self-efficacy to intervene. The authors^16^ emphasized the stigma of mental illness (i.e., negative stereotypes against people with mental illness) as potential causes to reduce gatekeepers’ decisions to intervene. Additionally, secondary traumatic stress resulting from interventions may reduce the likelihood of acting in future interventions. Lawson et al.^21^ noted that “educators need a trauma-informed literacy that enables self-care, facilitates and safeguards interactions with trauma-impacted students and colleagues, and paves the way for expanded school improvement models” (p. 421). As such, we deemed it important to consider self-care practices, including mindfulness and knowledge about self-care^21^ in gatekeeper training.

We identified a holistic approach for gatekeeper training that includes knowledge about suicide prevention, mental issues, and self-care. Specifically, EBP curricula address students’ behavioral and mental health needs through five intervention modules: Children’s Mental Health (CMH), Trauma Informed Classrooms (TIC), Youth Suicide Prevention (YSP), Trauma 102 (T102), and Self-Care and Mindfulness (SCM). EBP training is recognized by the Texas Education Agency as training that fulfills the requirements for mental health and suicide prevention training. Briefly, EBP interventions are:

- *Children’s Mental Health* (CMH): defines mental health, symptoms of mental health disorders, and presents strategies for empathetic conversations.
- *Trauma Informed Classrooms* (TIC): examines the effects of trauma on brain development, creating trauma informed spaces, and addresses grief in childhood.
- *Youth Suicide Prevention* (YSP): presents myths and facts of suicide and intervention strategies for youth with suicidal ideation.
- *Advanced Trauma* (T102): strategies to create trauma informed classrooms and campuses, including impacts of cultural awareness, equity, and racial/historical trauma in the classroom.
- *Self-Care and Mindfulness* (SCM): addresses employee burnout, establishing achievable SMART goals, and developing self-care plans.

The purpose of this program evaluation was to assess the effectiveness of EBP training (i.e., five EBP interventions). We measured effectiveness as changes in training participants’ perceptions and/or knowledge about youth behavioral and mental health issues and suicide prevention. Kirkpatrick and Kirkpatrick’s^22^ four-level approach (i.e., reaction, learning, behavior, and results) is a popular model for evaluating learner outcomes in training programs^23^. We applied the learning level (i.e., learner outcomes) to measure changes in learners’ perceptions and/or knowledge. Kirkpatrick and Kirkpatrick’s^22^ model does not account for intervening variables such as trainer’s expertise or learner motivation or previous knowledge. Therefore, results are confined solely to training program participants.

## Methods

A longitudinal trend survey^24^ was used because it permits data collection at different points. The target population included school personnel (e.g., teachers, principals, counselors), parents, and community members (i.e., child-serving agencies in school districts) affiliated with public or private schools (Table 1).

**Table 1:** Participants at Intervention Training (EBP Training), 2019–2020.

EBP Interventions (Topics)	2019	2020	Total
Children’s Mental Health (CMH)	3896	703	4599
Trauma-Informed Classrooms (TIC)	3055	1188	4243
Youth Suicide Prevention (YSP)	2625	799	3424
Trauma 102 (T102)	–	727	727
Self-Care and Mindfulness (SCM)	4	885	889

*Note*. Participants represented 28 Texas public school districts, four charter school systems, seven special needs schools, four private schools, 28 behavioral health providers, 26 community education and advocacy groups, 11 public entities, 20 child-serving organizations, two funders and four institutions of higher learning.

The CSBH notified selected school districts (general announcements in school newsletters) about its professional development programs 1–2 months in advance (typically in mid-spring) to coincide with school districts’ end-of-year continuing education programs. An online consent script informed each participant that anonymous responses would be used in group format for technical reports and publications; consent required acknowledgement (I Agree) before entry to the training surveys. A combination of instruments were used for data collection. Pre-intervention data were collected during training session registration for the CMH, SCM, T102 interventions. Immediately after those sessions, participants answered post-intervention questions that were the same as the pre questions. In training settings where changes in perceptions were sought, retrospective only post-intervention questions were administered immediately after training to reduce response-shift bias. The retrospective post-then-pretest design takes less time to administer and potentially eliminates participants’ over/underestimation of knowledge, skills, or experiences that may be interpreted differently during a pretest/post-test design, in which the pretest may alert participants to potential training topics. Not all participants completed all assessments (i.e., pretest/post-test or retrospective “before and after” questions); therefore, only paired data are reported herein.

Multiple purposive (non-probability) samples were drawn from the EBP interventions (i.e., target population) for matching data only on the variables of interest. Due to space limitations, primary demographics are in Table 2 (missing and ‘decline to answer’ data were excluded on a case-by-case basis). Each intervention included more than 400 participants. A post-power analysis using G*Power 3.1.9.7. Faul et al.^25^ indicated a sample of 400 was large enough to detect within-subject interactions (i.e., post hoc matched pairs; α = 0.05) as small as *d* = 0.2 with 98% power. Participants, across all EBP modules, were characterized as female (~ 82%), white (~ 58%), teachers (~ 76%), who were 36–45 years old (~ 37%) (Table 2). They were 41 years (*SD* = 10.90) old.

**Table 2:** Participants’ Characteristics from EBP Intervention Trainings (2019–2020).

Variables*	CMH (*N* = 1066)		TIC (*N* = 2133)		YSP (*N* = 1673)		T102 (*N* = 428)		SCM (*N* = 401)	
	*n*	%	*n*	%	*n*	%	*n*	%	*n*	%
Sex^a^										
Female	618	77.54	754	78.87	747	80.32	379	91.55	340	89.01
Male	179	22.46	202	21.13	183	19.68	35	8.45	42	10.99
Race^b^										
White	430	59.39	527	62.66	545	63.89	213	58.20	22	10.78
Hispanic	163	22.51	175	20.81	201	23.56	92	25.14	86	42.16
Black	131	18.09	139	16.53	107	12.54	61	16.67	96	47.06
Profession										
Teacher	792	86.18	1360	77.32	1092	76.36	215	53.22	239	64.25
Paraprofessional	63	6.86	178	10.12	113	7.90	36	8.91	49	13.17
Counselor	7	0.76	115	6.54	109	7.62	109	26.98	47	12.63
Other Staff^c^	57	6.20	106	6.03	116	8.11	44	10.89	37	9.95
Age										
26–35	254	38.48	279	37.25	283	36.80	101	29.11	99	34.02
36–45	244	36.97	262	34.98	274	35.63	154	44.38	116	39.86
46–55	162	24.55	208	27.77	212	27.57	92	26.51	76	26.12

*Self-reported gender, race or ethnicity, age, and profession. ^a^ Responses included Decline to answer or Other. ^b^ Responses included Asian, Two or more races, American Indian or Alaska Native and Native Hawaiian or Other Pacific Islander. ^c^ Other Staff included coaches, librarians, school nurses, behavioral health specialists, etc.

The professional development activities and data reported herein are not considered research involving human subjects as defined by DHHS and/or FDA regulations. This study was exempt by an Institutional Review Board at Texas A&M University; it is a program evaluation.

### Training intervention: emotional backpack project (EBP) training

The CSBH provided more than 150 training sessions, using EBP interventions in selected Texas school districts from 2019 to 2020. The EBP is a year-long, train-the-trainer program. MHAGH’s train-the-trainer workshops lasted nine hours with about 1.5 h/topic. After EBP training, trainees returned to their respective schools to teach other school staff, using EBP intervention materials, about youth behavioral and mental health needs.

In person training was delivered in schools before the novel Coronavirus 2019 lockdown and virtual training was delivered after March 2020. Three MHAGH training specialists led the in-person and virtual workshops. Training methods included experiential activities such as role play simulation, problem solving scenarios, and small group discussion about youth mental health issues.

### Instrument and data collection

Perceptions and knowledge of EBP interventions were collected with instruments based on Kirkpatrick and Kirkpatrick’s^22^ second level of evaluation (i.e., learning); all instruments used Likert-type, 5-point scales (1 = strongly disagree, 5 = strongly agree; 1 = low, 5 = high; 1 = very uncomfortable, 5 = very comfortable; 1 = poor, 5 = excellent; 1 = never, 5 = always). Concerning perceptions, the CMH intervention included five pre/post items rated on a 5-point scale (strongly disagree to strongly agree). Cronbach’s alphas were 0.92 (pre) and 0.94 (post); both were highly reliable. Five retrospective post-then-pre items were rated with 5-point scales (poor to excellent; very uncomfortable to very comfortable). Cronbach’s alphas were 0.80 (before) and 0.79 (after), also deemed reliable. The TIC intervention used retrospective post-then-pre items with 5-point scales (low to high). Cronbach’s alphas were 0.89 (before) and 0.92 (after); deemed highly reliable. The YSP intervention included eight items (retrospective post-then-pre) with a 5-point scale (poor to excellent). Cronbach’s alphas were 0.93 (before and after); deemed highly reliable. T102 intervention included eight items for knowledge and comfort levels (retrospective post-then-pre) on a 5-point scale (poor to excellent). Cronbach’s alphas were 0.95 (before) and 0.97 (after); deemed highly reliable. The SCM intervention had three pre/post and ten retrospective post-then-pre items for knowledge and comfort on 5-point scales (never to always; poor to excellent). Cronbach’s alphas were 0.74 (pretest) and 0.97 (post-test) and 0.84 (before) and 0.95 (after) in retrospective post-then-pre items; all were highly reliable.

Knowledge was tested with questions from the EBP curricula. Twelve pre/post questions in T102 and 11 pre/post SCM knowledge questions were posed as single (4-item multiple-choice) or multiple response (check all correct) items. Testing effect threats were minimized by randomizing question and response order^26^. Participants’ responses were dichotomously coded (0 = incorrect, 1 = correct). Kuder-Richardson 20 (KR20) reliability coefficients were used because the KR20 appropriately determines internal consistency of measurements with dichotomous data^24^. KR20 for T102 was 0.71 (pre) and 0.64 (post); SCM yielded reliability 0.47 (pre) and 0.58 (post). Reliability was attributed to heterogeneity of items and item discrimination^27^, and limited number of questions. Results should not be generalized beyond the sample when KR20 scores are less than 0.60^28^. Knowledge results represent those in MHAGH’s EBP training.

Participants demographic characteristics were recorded during training session registration; not all participants provided demographic information in each session. All valid paired data were assigned unique numbers and personal information was removed before analyses. Descriptive and inferential statistics described the data. Alpha was 0.05 a-priori to determine if significant differences existed in pre/post perceptions or knowledge. Non-parametric tests (Friedman, McNemar) were used because analyses were conducted on individual Likert-type items that were not assumed to be on continuous scales and cannot be summed to represent EBP constructs or concepts.

### Ethics declaration

All experimental protocols were reviewed by the Texas A&M University Institutional Review Board (#IRB2019-1309). All methods were carried out in accordance with relevant guidelines and regulations. The Texas A&M University Institutional Review Board determined this activity was a program evaluation and not research involving human subjects as defined by DHHS and FDA regulations.

### Consent to participate

Informed consent was obtained from all subjects and/or their legal guardian(s) by acknowledging agreement with an online consent script before accessing online surveys. The Texas A&M University Institutional Review Board waived the process to document consent because this study did not involve more than minimal risk.

## Results

### Changes in perceptions of the EBP intervention

#### CMH intervention

Friedman tests revealed significant changes in respondents’ (*n* = 1065) perceptions of mental illnesses involving changes in health (Table 3). Perceptions changed from agree (*Md*_pre_ = 4.0) to strongly agree (*Md*_post_ = 5.0) after the CMH intervention. Respondents’ comfort levels significantly increased from good (*Md*_before_ = 3.0) to very good (*Md*_after_ = 4.0) for understanding whether a student may be developing a mental health challenge, and from comfortable (*Md*_before_ = 4.0) to very comfortable (*Md*_after_ = 5.0) for approaching the student, school support staff, or administrators to discuss students’ mental health issues. Effect size magnitude was interpreted using *W*_*Kendall*_, based on Cohen’s benchmarks^29^. Negligible (≤ 0.1), moderate (0.3), and large (≥ 0.5) effects were found.

**Table 3:** Significance and Effect Sizes for Changes in CMH Perceptions, 2019–2020 (n = 1063).

Statements^a^ (Pre/Post)	Medians		*df*	*χ*^2d^	*W*^e^
	Pre	Post			
Mental illnesses are health conditions involving significant changes in… thinking	4	5	1	77.13*	0.07
…emotions	4	5	1	44.06*	0.04
…behavior	4	5	1	66.96*	0.06
…a combination (thinking, emotions, and behavior)	5	5	1	48.32*	0.05
Early adverse life experiences can be a strong risk factor for developing mental illness	4	5	1	83.54*	0.08
Statements (Retrospective post-then-pre)	Before	After			
Understanding whether a student may be developing a mental health challenge^b^	3	4	1	684.06*	0.64
Comfort level when approaching…^c^					
A student you are concerned about	4	5	1	444.57*	0.42
Parent or guardian of a student	4	4	1	513.87*	0.48
School support staff with your student concerns	4	5	1	347.50*	0.33
School administrators with your student concerns	4	5	1	336.75*	0.32

^a ^Scale: strongly disagree (1), disagree (2), uncertain (3), agree (4) and strongly agree (5). ^b^ Scale: poor (1), fair (2), good (3), very good (4) and excellent (5). ^c^ Scale: very uncomfortable (1), uncomfortable (2), neither comfortable or uncomfortable (3), comfortable (4) and very comfortable (5). ^d^ Friedman test. ^e^ Kendall’s Coefficient of Concordance uses Cohen’s^29^ explanations of effect sizes, including 0.1 (small), 0.3 (moderate) and ≥ 0.5 (large).

*p < 0.001.

#### TIC intervention

Friedman tests revealed significant increases in respondents’ (*n* = 1215) median rankings (Table 4) for self-perceived understanding of the influence of trauma on learning, increasing from average (*Md*_before_ = 3.0) to high (*Md*_after_ = 5.0), and knowledge of trauma and/or grief informed classrooms, which increased from average (*Md*_before_ = 3.0) to above average (*Md*_after_ = 4.0). Large (≥ 0.5) effect sizes were noted.

**Table 4:** Significance and Effect Sizes for Changes in TIC Perceptions, 2019–2020 (n = 1215).

Statements^a^ (Retrospective post-then-pre)	Medians		*df*	*χ*^2b^	*W*^c^
	Before	After			
Understanding of the influence of trauma on learning	3	5	1	812.17*	0.67
Knowledge of trauma informed classrooms	3	4	1	886.11*	0.73
Knowledge of grief informed classrooms	3	4	1	845.87*	0.70

^a ^Scale: low (1), below average (2), average (3), above average (4) and high (5). ^b^ Friedman test. ^c^ Kendall’s Coefficient of Concordance uses Cohen’s^29^ effect size; ≥ 0.5 (large).

*p < 0.001.

#### YSP intervention

Friedman tests revealed significant changes (Table 5) in respondents’ (*n* = 1160) perceived knowledge of the actions to take when a student is at risk of suicide and the signs and symptoms of suicide risk in students. Both items changed from good (*Md*_before_ = 3.0) to very good (*Md*_after_ = 4.0) after the YSP intervention. Confidence in approaching a student, helping a student, asking a student about suicide, and approaching school counselors, administrators, or parents/guardians about students at risk of suicide changed from good (*Md*_before_ = 3.0) to very good (*Md*_after_ = 4.0). Large (≥ 0.5) effect sizes were noted.

**Table 5:** Significance and effect sizes for changes in YSP perceptions, 2019–2020 (n = 1160).

Statements^a^ (Retrospective post-then-pre)	Medians		*df*	*χ*^2b^	*W*^c^
	Before	After			
Knowledge of…					
The actions to take if a student is at-risk of suicide	3	4	1	858.54*	0.74
Signs and symptoms of suicide risk in students	3	4	1	853.04*	0.74
Confidence in…					
Approaching a student at risk of suicide	3	4	1	832.04*	0.72
Helping a student at risk of suicide	3	4	1	834.08*	0.72
Asking a student about suicide	3	4	1	848.07*	0.73
Approaching a school counselor about a student at risk	3	4	1	673.14*	0.58
Approaching an administrator about a student at risk	3	4	1	654.15*	0.56
Approaching a parent/guardian about a student at risk	3	4	1	799.02*	0.69

^a ^Scale: poor (1), to fair (2), good (3), very good (4) and excellent (5). ^b^ Friedman test. ^c^ Kendall’s Coefficient of Concordance uses Cohen’s^29^ effect size; ≥ 0.5 (large).

*p < 0.001.

#### T102 intervention

Friedman tests revealed (Table 6) significant increases in respondents’ (*n* = 428) median rankings for self-perceived knowledge of the cultural considerations of trauma, historical trauma, racial trauma, implicit bias, and equity; all increased from good (*Md*_before_ = 3.0) to very good (*Md*_after_ = 4.0) after the T102 intervention training in 2020. Comfort levels for responding to students with trauma, historical trauma, and/or racial trauma increased significantly from good to very good. Moderate (0.3) to large (≥ 0.5) effect sizes were noted.

**Table 6:** Significance and effect sizes for changes in T102 perceptions, 2020 (n = 428).

Statements^a^ (Retrospective post-then-pre)	Medians		*df*	*χ*^2b^	*W*^c^
	Before	After			
Knowledge of…					
The cultural considerations of trauma	3	4	1	227.50*	0.53
Historical trauma	3	4	1	251.13*	0.59
Racial trauma	3	4	1	211.59*	0.50
Implicit bias	3	4	1	234.47*	0.55
Equity	3	4	1	198.36*	0.47
Comfort level for responding to students with…					
Trauma	3	4	1	163.76*	0.38
Historical trauma	3	4	1	195.39*	0.46
Racial trauma	3	4	1	207.62*	0.49

^a ^Scale: poor (1), fair (2), good (3), very good (4) and excellent (5). ^b^ Friedman test. ^c^ Kendall’s Coefficient of Concordance uses Cohen’s^29^ effect sizes from 0.3 (moderate) to ≥ 0.5 (large).

*p < 0.001.

#### SCM intervention

Friedman tests revealed (Table 7) significant differences (χ^2^(1) = 127.52, *p* = 0.001) in respondents’ (*n* = 401) median rankings for engaging in self-care, although it was ranked as often (*Md* = 4.0) in pre/post-intervention. Moderate (0.3) effects were found in respondents’ perceived engagement in mindfulness practice and practicing mindfulness with students. Both items increased from sometimes (*Md*_pre_ = 3.0) to often (*Md*_post_ = 4.0). Respondents’ perceived knowledge and understanding increased significantly (*p* < 0.001) from good to very good for incorporating selfcare into daily routines, use of SMART goals in self-care, research that supports mindfulness, how to be attentive and aware and knowledge of practices to help oneself be present and attentive increased after the SCM intervention. Self-perceived knowledge of the impact stress has on health and how self-care can improve stress were rated as very good (*Md* = 4.0), before and after training. Respondents’ knowledge and understanding of the role of the vagus nerve, as described in EBP training materials, increased significantly (χ^2^(1) = 200.84, *p* = 0.001) from fair to good after the SCM intervention.

**Table 7:** Significance and Effect Sizes for Changes in SCM Perceptions, 2020 (n = 401).

Statements^a^ (Pre/Post)	Medians		*df*	*χ*^2c^	*W*^d^
	Pre	Post			
How often do you…					
Engage in self-care?	4	4	1	127.52*	0.33
Engage in mindfulness practice?	3	4	1	179.86*	0.45
Practice mindfulness with students?	3	4	1	167.54*	0.42
Statements^b^ (Retrospective post-then-pre)	Before	After			
Knowledge and understanding of…					
How to incorporate selfcare into daily life	3	4	1	189.24*	0.47
Use SMART goals in self-care	3	4	1	216.29*	0.54
The impact stress has on health	4	4	1	131.96*	0.33
How self-care can improve stress	4	4	1	142.10*	0.36
Research that supports mindfulness	3	4	1	180.84*	0.45
The role of the vagus nerve	2	3	1	200.84*	0.50
How to pay attention	3	4	1	158.33*	0.40
How to increase my awareness	3	4	1	183.21*	0.46
Practices to help me be present	3	4	1	195.41*	0.49
Practices to help me pay attention	4	4	1	186.12*	0.47

^a ^Scale: never (1), rarely (2), sometimes (3), often (4) and always (5). ^b^ Scale: poor (1), fair (2), good (3), very good (4) and excellent (5). ^c^ Friedman test. ^d^ Kendall’s Coefficient of Concordance uses Cohen’s^29^ effect sizes from 0.3 (moderate) to ≥ 0.5 (large).

*p < 0.001.

### Changes in knowledge

#### T102 intervention

An exact McNemar’s test revealed (Table 8) significant increases in proportions (*n* = 428) of correct responses for trauma-informed schools are consistent, predictable, positive (*p* = 0.001) and safe (*p* = 0.01). Knowledge gains ranged from ~ 4 to ~ 29%. Significantly more correct responses were chosen for “trauma informed educators foster cultural awareness and promote equity” (*p* = 0.001) after the T102 training. Knowledge gains ranged from ~ 5 to ~ 7%.

**Table 8:** Frequencies and Chi-Square Results for Correct T102 Knowledge Items, 2020 (n = 428).

Questions	Pre		Post		*χ*^2a^	*p*
	*n*	%	*n*	%		
Trauma informed schools are						
consistent	375	87.6	418	97.7	36.00*	0.00
Inflexible^b^	411	96.0	417	97.4	1.79	0.18^c^
Predictable	205	47.9	328	76.6	104.08*	0.00
Positive	363	84.8	410	95.8	38.47*	0.00
Unstructured^b^	417	97.4	423	98.8	2.50	0.11^c^
Safe	386	90.2	405	94.6	6.89*	0.01
Trauma informed educators						
Provide therapy to students^b^	318	74.3	327	76.4	1.26	0.26^c^
Show empathy	414	96.7	419	97.9	0.84	0.36^c^
Do not set boundaries^b^	396	92.5	399	93.2	0.19	0.66^c^
Foster cultural awareness	379	88.6	402	93.9	10.30*	0.00
Promote equity	382	89.3	413	96.5	20.00*	0.00
Ask students about the details of their trauma^b^	358	83.6	355	82.9	0.07	0.79^c^

^a ^McNemar test. ^b^ Incorrect. ^c^ Binomial distribution.

*p < 0.05.

#### SCM intervention

An exact McNemar’s test showed (Table 9) significant (*p* = 0.001) increases (8.5%) in correct responses for the post-intervention (*n* = 401) item “scholarly research finds that mindfulness practice increases attention.” Significantly (*p* = 0.001) more correct responses were selected for post-intervention items, “Diaphragmatic breathing activates [the vagus nerve]” (12% increase), and “What does the S in SMART goals mean? [Specific—a goal must be focused]” (~ 14% increase).

**Table 9:** Frequencies and Chi-Square Results for Correct SCM Knowledge Items, 2020 (n = 401).

Questions	Pre		Post		*χ*^2a^	*p*
	*n*	%	*n*	%		
Research shows mindfulness practice						
Decreases stress and anxiety	380	94.8	367	91.5	3.20	0.07
Increases attention	321	80.0	355	88.5	13.28*	0.00
Increases IQ	189	47.1	183	45.6	0.25	0.62
Improves interpersonal relationships	348	86.8	343	85.5	0.23	0.64
Mindfulness helps us calm the limbic system so we can connect with our [higher-level functions]	154	38.4	157	39.2	0.03	0.86
The proper technique for a deep breath is to [extend] your stomach on inhale and [contract] it on exhale	184	45.9	200	49.9	2.30	0.13
Diaphragmatic breathing activates [the vagus nerve]	199	49.6	247	61.6	16.74*	0.00
What does the S in SMART goals mean? [Specific—a goal must be focused]	279	69.6	334	83.3	28.87*	0.00
Incorporating consistent mindfulness practice into your curriculum is a great way to promote [social] and emotional learning in your classroom	182	45.4	189	47.1	0.40	0.53
Mindfulness draws on an explicit value system that emphasizes wholesome attitudes including all the following EXCEPT [formality]	311	77.5	298	74.3	1.43	0.23
Youth are prone to making unwise decisions when an overstimulated limbic system causes decreased thought processes in the [prefrontal cortex]	207	51.6	212	52.9	0.11	0.74

^a^McNemar test.

*p < 0.001.

## Discussion

We evaluated the EBP training program in terms of changing school personnel perceptions (beliefs and attitudes) and knowledge about youth behavioral and mental health issues and suicide prevention. After training, respondents reported significant improvements in mental health literacy and knowledge of students’ behavioral and mental health issues. MHAGH’s trainings were “successful in meeting educators’ needs as well as enhancing educators’ confidence in effectively addressing youth mental health needs in schools” (^12^, p. 221). This study supports the notion that professional development for school personnel helping students cope with behavioral and mental health issues is time well spent.

We affirmed previous work^13^ about educators’ increased knowledge and changed beliefs about students’ mental health issues and increased their confidence in addressing such issues. Changes in beliefs about students’ mental health may lead to broader understanding of student behavior and academic achievement. We found significant increases in educators’ confidence to help students at risk of suicide. EBP intervention training could help educators learn how to detect earlier those students with suicidal ideation, which may lead to earlier treatment from mental healthcare professionals. However, increased awareness of students’ mental health issues and/or confidence to help students at risk of suicide does not specifically translate into active prevention of youth suicide^8,15,16^. Future post-training indicators of participants’ active practice of the lessons learned from EBP intervention training are needed to understand better if this training produces observable effects in preventing youth suicide.

Consistent with previous studies^10–13^, participants’ perceived knowledge of students’ behavioral and mental health issues increased after training. Self-care practices, including mindfulness and knowledge about self-care, are important components to reduce secondary traumatic stress^21^. Post-intervention results showed participants felt very good (confident) about approaching school counselors, administrators, or students to discuss youths’ at-risk behaviors. These findings may reduce educators’ secondary traumatic stress by encouraging discussion about at-risk students and fostering self-care practices needed after traumatic experiences. These trends also lend themselves to earlier interventions and better outcomes.

Educators’ responses to students can be harmful or healing and may depend on the teacher’s mental and emotional state. We encourage educators to have short- and long-term self-care plans to remain clear minded when responding to disruptive students. Lawson et al.^21^ noted that educators who interacted with traumatized students were themselves vulnerable to secondary traumatic stress. Dye et al.^30^ found that mindfulness training significantly helped counselors’ relaxation strategies. We advocate for schools integrating mindfulness practices into daily routines to support staff’s mental health and wellness.

After training, school-based mental health professionals’ confidence or comfort levels for approaching students to discuss mental health needs increased. School-based mental health professionals and administrators are underutilized resources in promoting positive mental health in schools^17–19^. Our interventions were attended by non-teachers, sometimes as much as 33% were school personnel from outside the classroom; increasing those participants’ confidence and comfort is needed when approaching students to discuss mental health needs. If more school personnel were confident in approaching students, those students might find support systems sooner, considering that youth (< 18 years) normally do not seek support for mental health^15^.

Participants were comfortable in approaching parents or guardians of students they were concerned about following the CMH intervention training and very comfortable approaching students or school support staff and administrators for the same reasons. We see a common, but potentially harmful gap in addressing students’ behavioral and mental health issues through a whole-community approach. Do teachers not discuss concerns about at-risk students with parents or guardians for fear of crossing a caregiver boundary? Hatton et al.^31^ reported secondary school teachers feared intervening with potentially suicidal students, while Ohrt et al.^32^ noted teachers’ training programs on mental health were missing communication skills building to interact with students with mental health issues. Our findings extend the lacking communication skills’ premise to include parents and/or guardians.

We believe less comfort in approaching parents or guardians was because of lacking communication skills or confidence as an advocate for student behavioral and mental health. It could be the result of unfamiliarity in discussing such matters with diverse families. Future training can build educators’ confidence and comfort in approaching parents or guardians by practicing improved communication skills. Although time-consuming, adequate training includes practicing communications, messaging, and listening skills. Role-play simulation, debriefing, and lessons learned reinforces communication strategies and increases teachers’ confidence in mental health training.

### Limitations

Although this report addressed outcomes of a program evaluation, a limitation existed in not having a control group to compare the impact of participation vs. non-participation in the EBP training. Future attempts should use experimental or quasi-experimental designs to measure the impact of professional development activities more effectively. Limitations exist in using retrospective post-then-pretest evaluation questions. Retrospective assessments might have introduced threats that interfered with measuring knowledge and/or confidence^33^. An improved program evaluation design will reduce or control most threats to internal validity. We used Kirkpatrick and Kirkpatrick’s^22^ program evaluation model, which was limited solely to program participants and cannot be generalized to other populations. Additionally, greater emphasis on tracking the number of school districts that participated in the EBP training, as well as number and type of school personnel attendees, will expand understanding of the true effectiveness of this professional development program. Finally, future investigations of EBP trainings should include measurements of post-training indicators such as teacher intervention, suicide data, and other school data (e.g., youth who self-report increased stressors following traumatic events) to expand understanding of the practical effectiveness of this mental health intervention and suicide prevention program.

## Conclusion

After EBP training, educators and school personnel reported having improved their knowledge and confidence in addressing students’ behavioral and mental health issues. Increased knowledge of mental illnesses can affect action because informed educators and school personnel can identify and refer students to mental health services. We recommend mental health training be conducted more frequently than one time annually and should include all school personnel. Training to build school personnel’s confidence in helping students with mental health issues and communication skills with primary caregivers is needed. It may help to study the implications of including cultural relevance in mental health training curricula to increase participants’ confidence for such roles. Confidence-boosting interventions can be designed for specific audiences (i.e., students, parents, educators, and school personnel) for use when communicating with at-risk students. Online intervention materials about students’ behavioral and mental health needs could help educators retain knowledge after in person or online training. Role-play simulation will improve educators’ confidence and communication skills for interacting with others to address students’ behavioral and mental health issues.

Students’ mental health issues were exacerbated during the novel Coronavirus 2019 (COVID-19) pandemic. Hill et al.^34^ found rates of suicide ideation and attempts were higher in 2020 compared to 2019, for youth aged 11 to 21 in a major Texas city. The onset of COVID-19 led to high unemployment, diminished income, and increased food and housing assistance needs^35^. These challenges compound trauma and toxic stress, leaving youth feeling unsafe, overwhelmed, and helpless. Educators and school personnel who are comfortable addressing such issues are crucial frontline responders for student mental health development. We advocate for increased school personnel training to build capacity for helping students cope with behavioral and mental issues in Texas.

## Data Availability

Datasets generated and analyzed during this study are available from the corresponding author on reasonable request.
